# Tuck-in Lamellar keratoplasty with an lenticule obtained by small incision lenticule extraction for treatment of Post- LASIK Ectasia

**DOI:** 10.1038/s41598-017-18201-4

**Published:** 2017-12-19

**Authors:** Yang Jiang, Ying Li, Shan Yang, Thomas Chengxuan Lu

**Affiliations:** 1 Department of Ophthalmology, Peking Union Medical College Hospital, Chinese Academy of Medical Sciences, Beijing, 100730 China; 2 St George Hospital, Gray Street, Kogarah, NSW 2217 Australia

## Abstract

Corneal ectasia is a rare but serious post-operative complication of LASIK. Our main aim was to describe and evaluate the efficacy and safety of tuck-in lamellar keratoplasty with an lenticule obtained by SMILE for treatment of Post LASIK Ectasia. Tuck-in lamellar keratoplasty was performed on three post-LASIK cornea ectasia patients (three eyes) with central corneal thickness less than 400 µm. Each patient was monitored for at least 12 months. Our primary outcomes measured pre and post-operatively included: Anterior segment optical coherence tomography(AS-OCT), corneal topography and monitored by slit-lamp microscopy. The mean total corneal thickness preoperatively and 1 day, 1 month, 3 months and 12 months post-op were 360.00 ± 32.07 µm, 590.00 ± 10.00 µm, 536.67 ± 11.54 µm, 523.33 ± 37.85 µm, 466.67 ± 41.63 µm. The mean lenticule implanted 1 day, 1 month, 3 months and 12 months post-op were 173.33 ± 41.63 µm,136.67 ± 25.16 µm, 133.33 ± 40.41 µm, 130.00 ± 17.32 µm. There was no evidence of immune rejection or other complications. Tuck-in lamellar keratoplasty with an lenticule obtained by SMILE seems to be a safe and alternative surgical approach in the treatment of post - LASIK cornea ectasia, especially for severe cases with cornea thickness less than 400 μm.

## Introduction

Corneal ectasia, a rare complication of laser *in situ* keratomileusis (LASIK), was first reported in 1998 and has an incidence of 0.04% to 0.66%^1^. Although the incidence is rare, its consequences may be devastating and induce severe myopia, irregular astigmatism, and reduction of best corrected visual acuity. With the increasing number of LASIK procedures, more cases of post-LASIK cornea ectasia are being reported. Reduced biomechanical strength of the cornea is thought to be the reason for the development of postoperative ectasia due to thinning of the underlying keratoconus or a residual stromal bed.

In our study, we describe tuck-in lamellar keratoplasty with an lenticule obtained by SMILE, and report the safety and efficacy of this approach through a case series.

## Materials and Methods

### Patients

This is a prospective study carried out on patients attending Peking Union Medical College Hospital, Chinese Academy of Medical Sciences from April 2014 to December 2016. The use of human tissue samples was approved by the ethics committee of the Institutional Ethics Committee of Chinese Academy of Medical Sciences, Peking Union Medical College Hospital, and the procedures used conformed to the tenets of the Declaration of Helsinki. Possible benefits and risks were explained to all patients. Informed consent was obtained from all patients. All experiments were performed in accordance with the relevant guidelines and regulations of the ethics committee of the Institutional Ethics Committee of Chinese Academy of Medical Sciences. No tissues were procured from prisoners. Corneal graft were procured from patients who underwent Smile surgery in Peking Union Medical College Hospital. All donors were tested for HIV, hepatitis B, hepatitis C and syphilis.

### Inclusion criteria

The inclusion criteria in this study were as follows: (1) post-LASIK ectasia (maximum K reading >50 D); (2) with documented clinical worsening and instrumental progression by an increase of 1.0 D or more in maximum K reading and reduction of corneal thickness at the thinnest point by 10 µm or more for at least 3 months; (3) Minimal corneal thickness <400 µm; (4) completely clear cornea without any other ocular or systemic disease; (5) aged 18–40 years.

### Exclusion criteria

The following conditions were considered as exclusion criteria in this study: (1) history of herpetic keratitis, concurrent corneal infections, or concomitant autoimmune diseases; (2) severe dry eye, acute hydrops, severe allergic conjunctivitis and diffuse central corneal opacity; (3) recent contact-lens users; (4) glaucoma, cataract, or vitreoretinal disorders, pregnancy or lactating; (5) mental illness.

### Preoperative examination

Three patients (3 eyes) were monitored for at least 12 months and were assessed using slit-lamp microscopy, corneal topography (Tomey, TMS-4, Japan), optical coherence tomography (Visante OCT, Carl Zeiss Meditec, Dublin, USA) and best corrected visual acuity (BCVA). Postoperative complications throughout the study period were recorded.

### Surgical Technique

Eligible donors underwent SMILE with the standard technique by the same surgeon using the VisuMax FS laser (Carl Zeiss Meditec, Jena, Germany). The cap thickness was 120 μm and the optical zone varied from 6.0 to 6.5 mm. After dissection of both anterior and posterior planes, the lenticule was extracted through a superior 4-mm incision. The lenticule was immediately transferred into sterile 0.9% sodium chloride solution (CR Double-Crane, Beijing, China) and prepared to be used. Implantation of the lenticule was performed immediately after the SMILE procedure. All surgeries were performed under peribulbar anesthesia (0.5% lidocaine).

**Figure 1:**
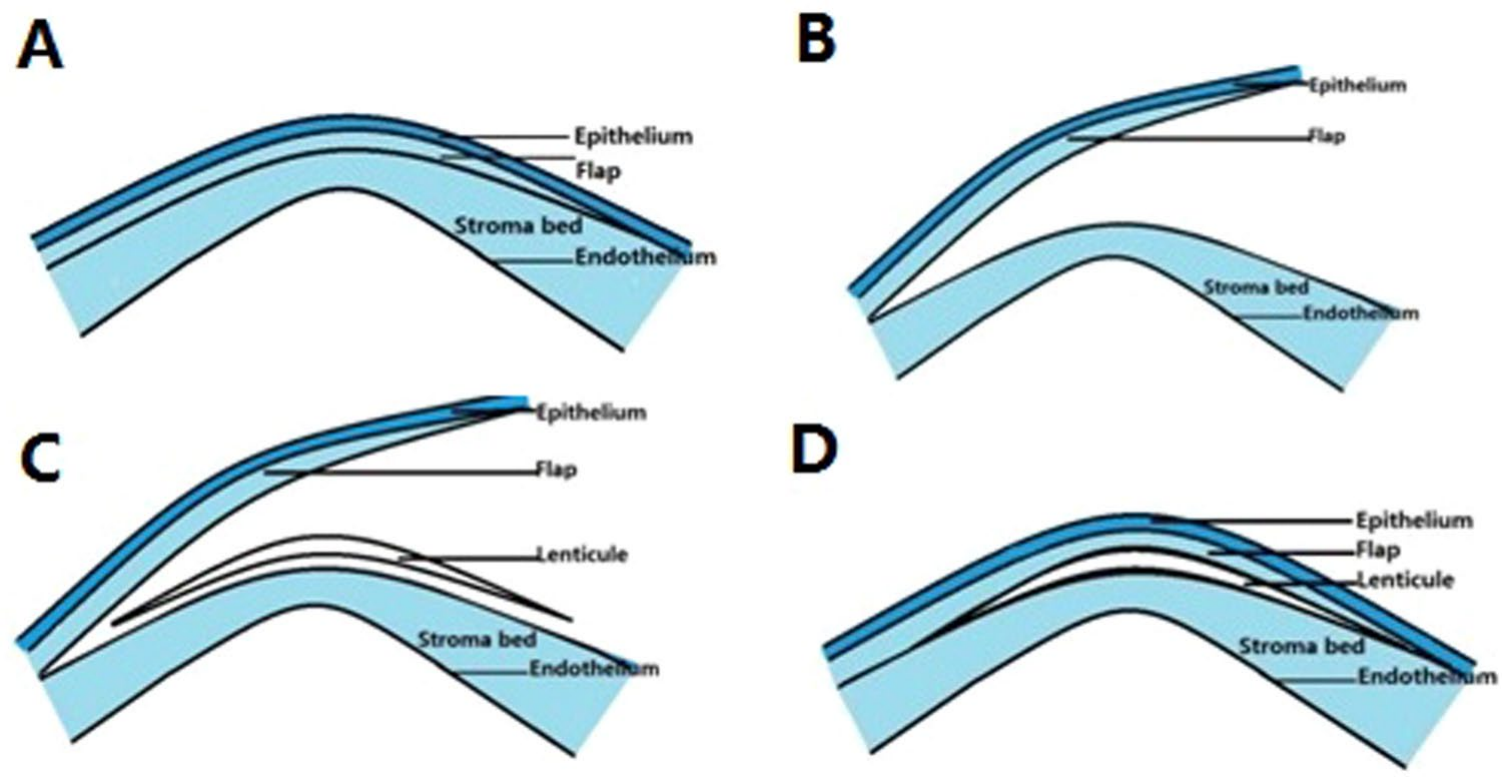
Tuck-in lamellar keratoplasty with an lenticule obtained by SMILE for treatment of post-LASIK Ectasia (**A**) Corneal conditions of the post-LASIK ectasia preoperatively. (**B**) The LASIK flap was carefully lifted up by pressing the central part of the cornea with a cotton-tipped applicator. (**C**) The corneal intrastromal lenticule was implanted on the conus cone between the LASIK flap and the stroma bed. (**D**) The LASIK flap was restored to the original position.

The LASIK flap was carefully lifted up by pressing the central part of the cornea with a cotton-tipped applicator. Few adhesions were encountered between the LASIK flap and the underlying stroma that were gently separated using a lamellar separator. The corneal intrastromal lenticule was implanted on the conus cone between the LASIK flap and the stroma bed. And then, the LASIK flap was restored to the original position(Fig. 1). Finally, one piece of bandage contact lens (Bausch and lomb, USA) was worn and 0.3% ofloxacin topical eye ointments (Santen, Osaka, Japan) were administered in the conjunctival sac.

### Perioperative Treatment

Preoperative management included topical 0.5% levofloxacin eye drops (four times a day; Santen, Japan). Postoperatively, topical steroid (1% prednisolone acetate; Allergan, Irvine, CA) was prescribed for 6 months four times a day. In addition, anti-biotic eye drops (0.3% Ofloxacin Eye Oitment; Sinqi; Shenyang, China) was administered four times daily during the first month post-op. Corneal crosslinking were suggested to be combined with this procedure to improve the biomechanical strength of the cornea.

### Perioperative Evaluation

Patients were examined postoperatively at day 1, months 1, 3, and 12. Slit-lamp microscopy was performed to check the corneal healing day 1 post-op. Assessments performed during the following post-op visits include: BCVA, slit-lamp microscopy, corneal topography and optical coherence tomography. Any complications would be recorded.

### Data Availability

The datasets generated during the current study are available from the corresponding author on reasonable request.

## Results

Three patients (3 eyes) were recruited, including 2 females and 1 male with a mean age of 28.0 ± 3 years (range, 25–31 years). The clinical characteristics of the patients are summarized in Table 1.

**Table 1:** Demographics and clinical findings of post-LASIK ectasia patients underwent tuck-in lamellar keratoplasty with an lenticule obtained by SMILE for treatment of post-LASIK Ectasia (*n* = 3).

Case No.	Gender/age	Eye	Initial Ks	Final Ks	Initial CCT	Final CCT
1	F/31	LE	50.88	54.37	380	500
2	M/28	RE	52.40	59.31	377	420
3	F/25	RE	51.01	57.70	323	480

F: female; M: male; LE: left eye; RE: right eye; Ks: K steep; CCT: central corneal thickness (µm).

**Figure 2:**
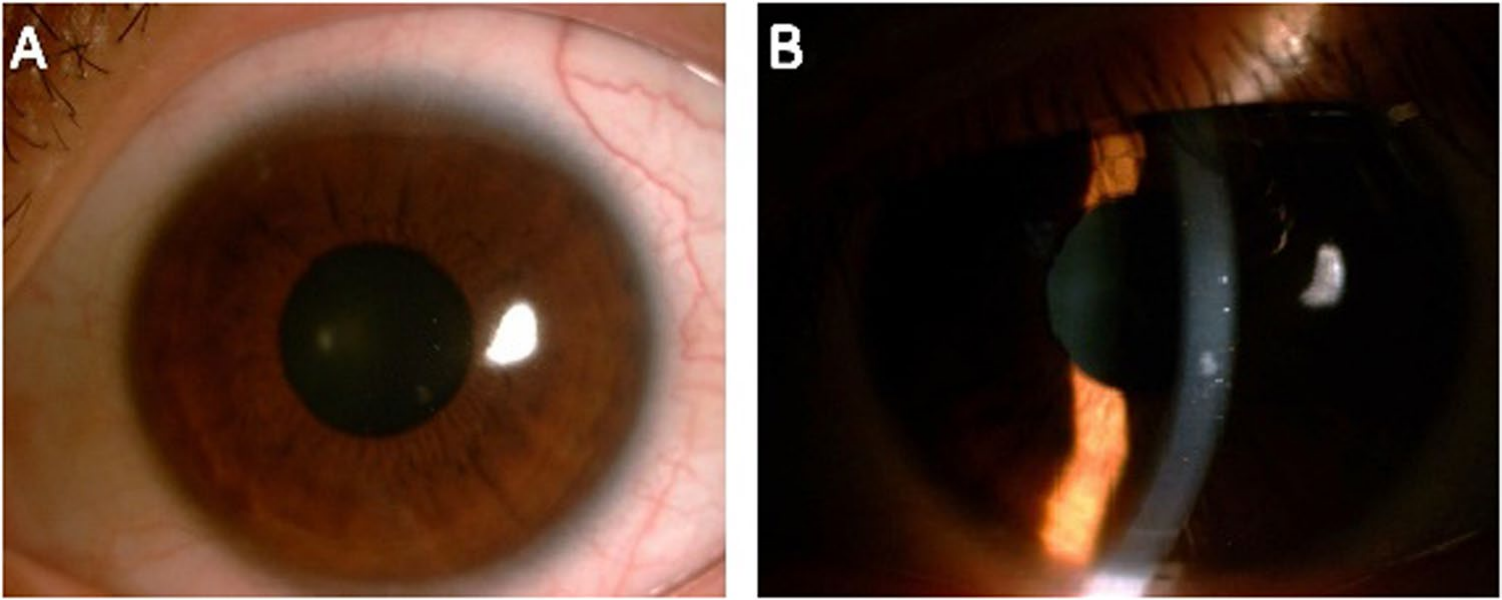
Corneal conditions of the post laser-assisted *in situ* keratomileusis (LASIK) ectasia patient at 12 months after tuck-in lamellar keratoplasty with an lenticule obtained by SMILE for treatment of post-LASIK Ectasia (patient 1). (**A**) The cornea was clear with no neovascularization or rejection. (**B**) The corneal lenticule was clear and there were no flap-related complications. The best corrected visual acuity improved from 0.3 preoperatively to 0.8 at 12 months after surgery.

**Figure 3:**
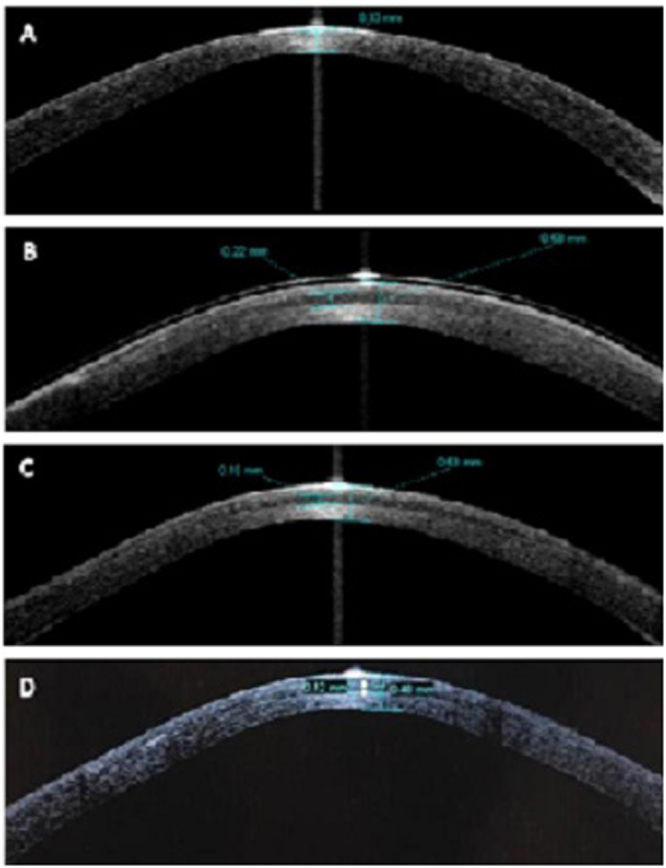
Optical coherence tomography images of the cornea in post laser-assisted *in situ* keratomileusis (LASIK) ectasia patient (patient 3). (**A**) Preoperatively, the residual corneal thickness was only 330 μm. (**B**) The total corneal thickness was 580 μm, while corneal lenticule thickness was 220 μm on the first day after surgery. The interface between the corneal lenticule and the stroma bed was clear. (**C**) The total corneal thickness was 530 μm, while corneal lenticule thickness was 160 μm in 2 weeks after the surgery. (**D**) The total corneal thickness was 480 μm, while corneal lenticule thickness was 120 μm at 12 months after the surgery. The interface between the corneal lenticule and the stroma bed became ambiguous.

At the final follow-up (12 months), all the grafts were clear (Fig. 2). The mean corneal thickness measured by AS-OCT preoperatively and 1 day, 1 month, 3 months and 12 months post-op were 360.00 ± 32.07 µm, 590.00 ± 10.00 µm, 536.67 ± 11.54 µm, 523.33 ± 37.85 µm, 466.67 ± 41.63 µm respectively. The mean corneal lenticule implanted measured by AS-OCT 1 day, 1 month, 3 months and 12 months post-op were 173.33 ± 41.63 µm,136.67 ± 25.16 µm, 133.33 ± 40.41 µm, 130.00 ± 17.32 µm respectively. Corneal and lenticular thickness initially decreased, but stabilized 12 months after the operation. Meanwhile, the interface between the corneal lenticule and the stroma bed became ambiguous (Fig. 3).

**Figure 4:**
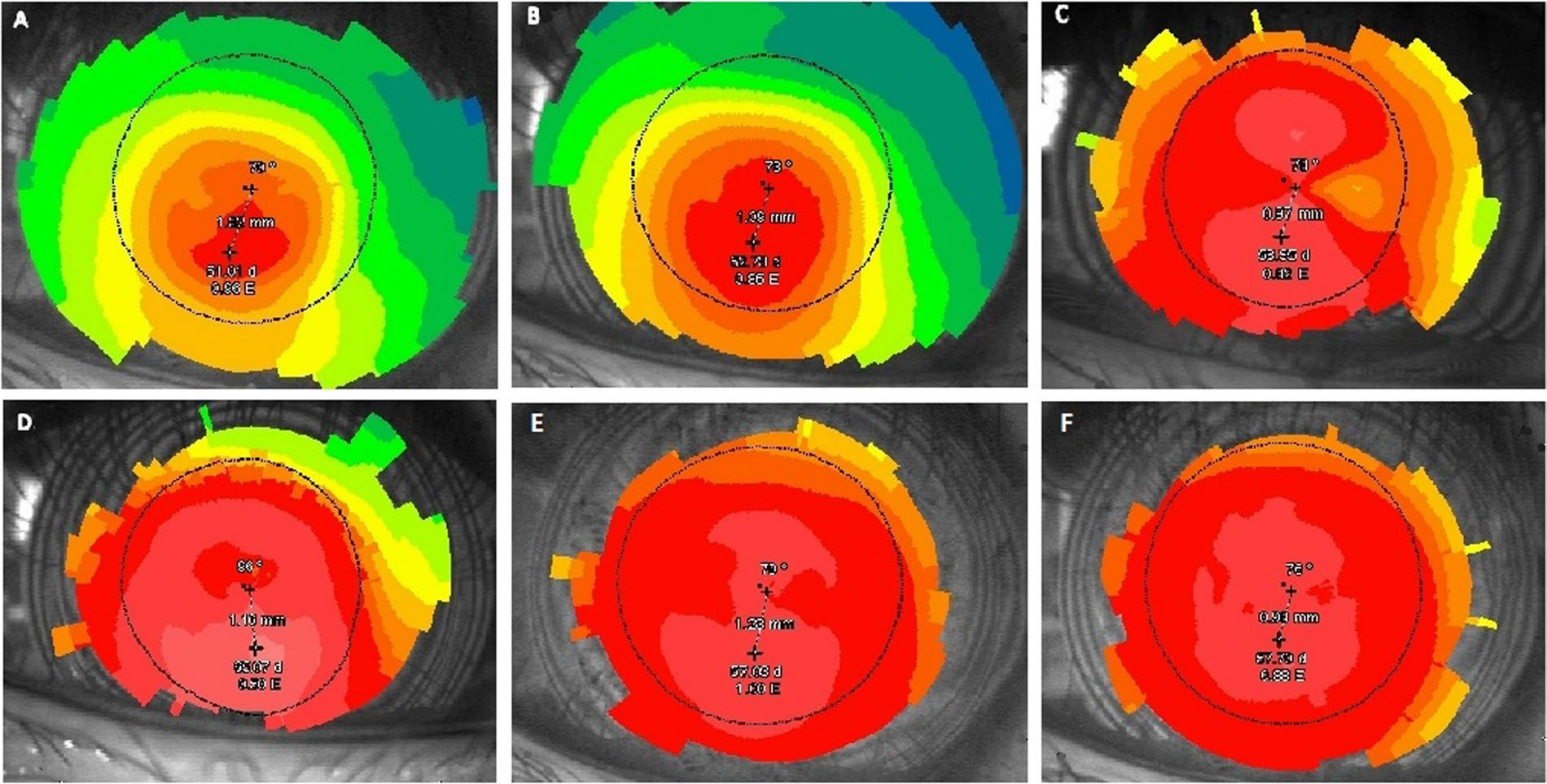
The topograghy images of the cornea in post laser-assisted *in situ* keratomileusis (LASIK) ectasia patient (patient 3). (**A**) Preoperatively, the max K value was 51.01. (**B**) After 3 months of observation, the max K value increased to 52.73. (**C**) The max K value was 58.35 one day after the surgery. (**D**) The max K value was 65.07 two weeks after the surgery. (**E**) The max K value was 57.03 at 3 months after the surgery. (**F**) The max K value was 57.70 at 12 months after the surgery.

The mean min K value measured by topograghy preoperatively and 1 day, 1month, 3months and 12 months postoperative was 44.86 ± 3.92, 48.83 ± 2.38, 50.63 ± 4.24, 49.71 ± 3.26 and 50.07 ± 3.43 respectively. The max K value increased significantly after the operation and then declined gradually (Fig. 4).

The biomechanical strength of the cornea in patient 1 was measured by Corvis®ST (OCULUS, Germany). The deformation amplitude (DA value) of the cornea 1 month and 3 months postoperatively was 0.999 mm and 1.052 mm respectively, which was within the range of healthy corneas DA value^2^ (range: 0.91 to 1.26 mm).

### Perioperative Complications

The mean follow-up period was 13.33 ± 0.57 months. No immune rejection or other complications occurred in any of the patients.

## Discussion

Corneal ectasia is a rare but severe complication of refractive surgery. The clinical signs of corneal ectasia include stromal thinning, anterior and posterior corneal steepening, a variable and frequently progressive increase in myopia, irregular astigmatism, corneal aberrations, followed by a consequent loss of corrected distance visual acuity (CDVA).

The treatment options for post-LASIK ectasia include contact lens fitting, intracorneal rings, collagen cross linking, deep anterior lamellar keratoplasty and penetrating keratoplasty. When ectasia progresses to the point where contact lenses or collagen cross linking could no longer provide useful vision or be able to enhance the cornea, surgical intervention would be considered. Penetrating keratoplasty is the most commonly performed surgical procedure for ectatic corneas, but is associated with complications including graft rejection, induced astigmatism, complications of intraocular surgery such as glaucoma, cataract formation, retinal detachment, cystoid macular edema, endophthalmitis, and expulsive hemorrhage. Historically, penetrating keratoplasty (PK) has been the standard of care in surgical management of keratoconus. More recently, numerous international retrospective studies have suggested that rates of PK for keratoconus are decreasing^3–8^. In recent years, Ferrara rings (Intrastromal Corneal Ring Segments, ICRS) have been introduced in South America and Europe to treat post-LASIK ectasia. Intraoperative complications include segment decentration, ICRS asymmetry, inadequate channel depth, superficial channel dissection with anterior Bowman layer perforation, and anterior chamber perforation^9^. Although ICRS is usually well tolerated, previous *in vitro* studies have described activation of keratocytes, accumulation of lipids in cells and new collagen formation after implantation. Several postoperative complications have been described^10^, including ring segment extrusion, corneal neovascularization, infectious keratitis, mild channel deposits around the ICRS, segment migration, and corneal melting. In the U.S. Food and Drug Administration phases II and III clinical trials of Intacs, segment removal was necessary in 4.68% of eyes^10^. Corneal crosslinking (CXL) with ultraviolet-A rays and riboflavin has been proposed as a method to prevent progression of keratoconus or post-LASIK ectasia. However, the corneal flap do not contribute to corneal structural integrity and might not enhance biomechanical strength of the ectatic cornea. Furthermore, the high intensity UVA radiation might lead to sub-basal nerve plexus injury, reducing Na/K-ATPase pump performance in corneal endothelium. Badawi reported that in post-LASIK patients, the mean endothelial cell density changed significantly from 2,698.40 ± 189.11 preoperative to 2,630.00 ± 193.09 at 3 months after the accelerated CXL (10 mW/cm^2^ for 9 minutes). The calculated endothelial cell loss rate was 2.53%^11^.

Discrepant histopathological, ultrastructural, and biochemical evidence suggests that the pathogenesis of post-LASIK ectasia and keratoconus could be different^12^. Post-LASIK ectasia might be considered the biologic equivalent of an interfiber disruption of composite materials. This occurs in biomechanically weakened corneas due to structural instability from excimer laser refractive surgery^13^.

Vajpayee RB *et al*.^14^ have described “tuck in” lamellar keratoplasty(TILK) in cases with corneal ectasias involving corneal periphery. In this technique, the donor lenticule has a full-thickness central part and a peripheral flange of partial thickness posterior stromal tissue. Then they performed TILK in cases with post-PKP ectasia involving the peripheral cornea and the graft-host junction^15^. In these cases, the donor lenticule has a full-thickness central part and a peripheral flange consisting of partial-thickness posterior corneal stroma. The central part of the full-thickness graft provides tectonic support to the central cornea, whereas the thin peripheral flange tucked into the intrastromal pocket provides tectonic support to the peripheral cornea beyond the previous graft–host junction. This technique provides better tectonic support to the peripheral ectatic cornea beyond the previous graft–host junction with no damage to the recipients’ limbal stem cells.

We believe this tuck in lamellar keratoplasty described by Vajpayee RB *et al*.^14,15^ can be a practical technique for treatment of corneal ectasia. Meanwhile, the shortage of corneal donors has always been bottlenecks in clinical treatment of corneal disease patients, especially in developing countries. From the best of our knowledge, this is the first report of tuck-in lamellar keratoplasty with an lenticule obtained by SMILE for treatment of post-LASIK ectasia. In all three patients, cornea and lenticule implanted thickness decreased post-operatively. This ceased at 12 month. The max K value also increased post- operatively. This is most likely due to the increase of total regular corneal curvature caused by the implantation of lenticule, as opposed to progression of ectasia associated with irregular anterior protrusion of the cornea. All the three patients (3 eyes) were monitored for at least 12 months, and all the grafts stayed clear. No immune rejection or any other complication occurred in any of the patients.

The corneal intrastromal lenticules extracted from SMILE are clear and high-quality. Donors who undergo refractive surgery are relatively young. Furthermore, the lenticules used in the procedure has diameter ranging from 6.0 to 6.5 mm, minimizing risk of donor rejection. Pradhan and Ganesh have separately reported their successful implantation of the lenticules extracted from the SMILE procedure to correct high hyperopia patients^16,17^. Our group have previously reported the successful use of this procedure in corneal ulcers and perforations^18^. No adverse side effects were observed in the follow-ups. It can also imply that the implantation of the extracted lenticules has potential clinical application to other ocular pathologies including aphakia, hypermetropia and presbyopia.

This study has described our preliminary experience of the procedure, providing technical methods, with quantitative results and progression of the corneal graft pre-and post-operatively. From our experience, it seems that this approach can be an alternative and safe treatment for cases of severe ectasia with cornea thickness less than 400μm, in which neither contact lens fitting nor conventional collagen cross linking would be suitable. We hope that this case series will contribute to this area of literature and encourage further discussion of this procedure within the scientific community.

Some technical points are worth mentioning. We suggest that OCT should be used preoperatively to evaluate the thickness of the cornea. Multiple pieces of lenticules could be used in severe cases. During the operation, LASIK flap should be lifted very carefully to prevent the fall-off of the corneal epithelium, which could lead to disastrous epithelial ingrowth postoperatively. Post-operatively one piece of bandage contact lens is suggested to be worn, which can reduce the risk of the epithelial ingrowth postoperatively and improve the wound healing.

This technique may not have improved the biomechanical strength of the cornea and there could still be continued ectasia of the stromal bed. Therefore the procedure sould be combined with crosslinking to reduce the risk of continued ectasia.

Some weaknesses to our case series include low number of patients and short mean follow up time of only 13 months. The Corvis results was only gathered for one patient. Hence no definitive conclusion can be drawn about possible improvement of the biomechanical strength of the cornea and cessation of the ectasia. Future research can examine the combination of this procedure with corneal crosslinking.

In summary, tuck-in lamellar keratoplasty with an lenticule obtained by SMILE can be an alternative and safe surgical approach for treatment of post-LASIK ectasia, especially for severe cases with cornea thickness less than 400μm. This procedure should be combined with crosslinking to reduce the risk of continued ectasia.
